# Recognition of H2AK119ub plays an important role in RSF1-regulated early *Xenopus* development

**DOI:** 10.3389/fcell.2023.1168643

**Published:** 2023-07-17

**Authors:** Saeid Mohammad Parast, Deli Yu, Chunxu Chen, Amanda J. Dickinson, Chenbei Chang, Hengbin Wang

**Affiliations:** ^1^ Department of Cell, Developmental and Integrative Biology, University of Alabama at Birmingham, Birmingham, AL, United States; ^2^ Department of Biochemistry and Molecular Genetics, University of Alabama at Birmingham, Birmingham, AL, United States; ^3^ Department of Biomedical Engineering, School of Engineering, Virginia Commonwealth University, Richmond, VA, United States; ^4^ Massey Cancer Center, Virginia Commonwealth University, Richmond, VA, United States; ^5^ Department of Biology, College of Humanities and Sciences, Virginia Commonwealth University, Richmond, VA, United States; ^6^ Department of Internal Medicine, Division of Hematology, Oncology and Palliative Care, School of Medicine, Virginia Commonwealth University, Richmond, VA, United States

**Keywords:** RSF1, PRC1, H2AK119ub, UAB domain, mesoderm, neural and neural crest, development, *Xenopus laevis*

## Abstract

Polycomb group (PcG) proteins are key regulators of gene expression and developmental programs via covalent modification of histones, but the factors that interpret histone modification marks to regulate embryogenesis are less studied. We previously identified Remodeling and Spacing Factor 1 (RSF1) as a reader of histone H2A lysine 119 ubiquitination (H2AK119ub), the histone mark deposited by Polycomb Repressive Complex 1 (PRC1). In the current study, we used *Xenopus laevis* as a model to investigate how RSF1 affects early embryonic development and whether recognition of H2AK119ub is important for the function of RSF1. We showed that knockdown of *Xenopus* RSF1, *rsf1*, not only induced gastrulation defects as reported previously, but specific targeted knockdown in prospective neural precursors induced neural and neural crest defects, with reductions of marker genes. In addition, similar to knockdown of PRC1 components in *Xenopus*, the anterior-posterior neural patterning was affected in *rsf1* knockdown embryos. Binding of H2AK119ub appeared to be crucial for *rsf1* function, as a construct with deletion of the UAB domain, which is required for RSF1 to recognize the H2AK119ub nucleosomes, failed to rescue *rsf1* morphant embryos and was less effective in interfering with early *Xenopus* development when ectopically expressed. Furthermore, ectopic deposition of H2AK119ub on the Smad2 target gene *gsc* using a *ring1a*-*smad2* fusion protein led to ectopic recruitment of RSF1. The fusion protein was inefficient in inducing mesodermal markers in the animal region or a secondary axis when expressed in the ventral tissues. Taken together, our results reveal that *rsf1* modulates similar developmental processes in early *Xenopus* embryos as components of PRC1 do, and that RSF1 acts at least partially through binding to the H2AK119ub mark via the UAB domain during development.

## 1 Introduction

Polycomb group (PcG) proteins were originally identified in *Drosophila* as repressors of *homeobox* (*Hox*) genes, mutations of which often cause homeotic transformation of posterior to anterior legs, characterized by a comb-like bristle phenotype (Lewis, 1978). Subsequently, homologues of *Drosophila* PcG proteins were identified in vertebrates and plants and emerged as key regulators of gene expression programs (Schuettengruber et al., 2017; Piunti and Shilatifard, 2021; Baile et al., 2022). In addition to regulating *Hox* genes and anterior-posterior axial identity, PcG proteins have been shown to influence expression of many central developmental regulators, including genes involved in signaling, such as components of the TGFβ, Wnt, and MAPK pathways, transcriptional controls, as well as metabolism (Brookes et al., 2012). PcG proteins and their target genes play pivotal roles in many aspects of cellular physiology, such as cell fate determination, epigenetic memory, cell lineage commitment, and *X* inactivation (Schuettengruber et al., 2017; Blackledge and Klose, 2021; Piunti and Shilatifard, 2021). Mutations in PcG proteins often lead to diverse developmental phenotypes in vertebrates, including humans, and have been causally linked to disease predisposition and progression, particularly cancer (Piunti and Shilatifard, 2021; Dong et al., 2022; German and Ellis, 2022).

The function of PcG proteins is achieved through forming multiple subunit complexes. Two major repressive complexes, Polycomb Repressive Complex 1 and 2, a.k.a. PRC1 and PRC2, have been studied extensively. PRC2 contains EZH2, SUZ12, and EED as core components and can methylate histone H3 at lysine 27 (H3K27me) (Margueron and Reinberg, 2011). PRC1 contains Ring1 (Ring1A in mouse), Ring2 (also called RNF2 or Ring1B in mouse), and Bmi1 as core components and mono-ubiquitinate histone H2A at lysine 119 (H2AK119ub) (de Napoles et al., 2004; Wang et al., 2004). These modifications on chromatin form a positive feed-back loop to enhance the repressive effects. H3K27me can facilitate PRC1 to target chromatin for H2AK119ub modification whereas H2AK119ub can also recruit PRC2 through the auxiliary subunit Jarid2 for H3K27me (Blackledge et al., 2014; Cooper et al., 2014; Kalb et al., 2014; Cooper et al., 2016). This positive feed-back, self-enhancement mechanism may help the establishment of large PcG repressive domains. These domains repress alternative cellular gene expression programs, which contribute to the stabilization and maintenance of cell type-specific programs (Boyer et al., 2006; Bracken et al., 2006; Lee et al., 2006). Deletion of PRC1 or PRC2 subunits in mouse embryonic stem cells (ESC) often results in the expression of differentiation-associated genes and causes these ESCs to differentiate spontaneously (Chamberlain et al., 2008; Loh et al., 2021; Thornton et al., 2014; Pasini et al., 2007; O'Carroll et al., 2001). Therefore, PcG proteins are critical for the maintenance of cell identity (German and Ellis, 2022; Loh and Veenstra, 2022).

One essential question regarding gene regulation by histone modifications is how these histone marks are recognized and interpreted by other cellular proteins to affect chromatin structures and gene expression programs. We addressed this question in our previous studies by identifying a H2AK119ub-binding protein, Remodeling and Spacing Factor 1 (RSF1), the large subunit of RSF complex (LeRoy et al., 1998; Zhang et al., 2017). RSF1 can recognize H2AK119ub nucleosomes through a previously uncharacterized region we designated as the ubiquitinated H2A binding (UAB) domain. The UAB domain, spanning amino acid 770-807, interacts with H2AK119ub nucleosomes in a bi-partition model through a ubiquitin-interacting motif at its middle region and an arginine-anchoring mechanism at its N-terminal region. We showed in previous work that RSF1 is required for H2AK119ub target gene silencing and helps maintain regularly spaced H2AK119ub nucleosome patterns at promoter regions (Zhang et al., 2017). We also discovered that Rsf1 is required for vertebrate development. Specifically, loss of Rsf1 interrupted gastrulation and mesodermal induction in *Xenopus laevis* (Zhang et al., 2017). However, the importance of recognizing H2AK119ub *via* the UAB domain in RSF1 function was not addressed in these studies. In addition, the role of RSF1 during vertebrate embryogenesis has not been analyzed in depth. In this study, we extended our previous work and further investigated the activities of Rsf1 during development using the *X. laevis* model. While our previous studies established a role for Rsf1 in early stages of development, our investigation here revealed that Rsf1 also regulated other tissue types later in development. Another goal of this study was to determine whether the RSF1 UAB domain is important in embryos as it is in cells. We were interested in determining whether RSF1 also mediates the repressive effects of H2AK119ub on gene expression during embryonic development. Our studies revealed that the PRC1-H2AK119ub-RSF1 system, which was established with previous biochemical and cell-based assays, is also operating during normal *Xenopus* development, and that the UAB domain is important for the function of RSF1 in this system.

## 2 Materials and methods

### 2.1 Embryo manipulations

The utilization of *X. laevis* was under the institutional IACUC protocol 21854 at the University of Alabama at Birmingham and IACUC protocol AD20261 at Virginia Commonwealth University. *X. laevis* embryos were obtained by *in vitro* fertilization and micro-injected with RNAs or MOs, as described in previous publications (Tien et al., 2021; Mohammad Parast and Chang, 2022). The human RSF1 or RSF1ΔUAB (fused with GFP) sequences were moved from the pCDNA3 to the pCS105 vector, making them amenable for *in vitro* RNA synthesis for expression in *Xenopus*. The *Xenopus ring1a* (*ring1a.L*, XB-Gene-6493980, NCBI accession # XM_041572851) sequence was PCR-cloned into the *EcoRI/XbaI* cut CS105 vector using the primers: forward: 5’-GGA​ATT​CAC​CAG​TTT​AAA​GAC​AAT​GGC-3’ and reverse: 5’-GCTCTAGA CTA​TTT​CTG​CTC​TTT​GGT-3’. Ring1A R to Q mutation was generated by PCR using the primers: forward: 5’-AGA​AGC​TGG​TGT​CCA​AGC​AAT​CCC​TAC​G-3’, reverse: 5’- TGG​CCG​TAG​GGA​TTG​CTT​GGA​CAC​CA-3’. The *Xenopus rnf2* (*rnf2.L*, XB-Gene-6538658, NCBI accession # XM_041589074) sequence was PCR-cloned into *EcoRI/XhoI* cut pCS105 vector using the primers: forward: 5’-GGA​ATT​CAC​CAT​GAA​TTG​CAT​CAG​CAT​GC-3’ and reverse 5’-CCG​CTC​GAG​TTA​TTT​GTG​CTC​CTT​GGT​G-3’. For constructing *ring1a-smad2*, *ring1a* sequence was PCR-amplified from pCS105-ring1a with primers: forward, 5’-GGA​ATT​CAC​CAG​TTT​AAA​GAC​AAT​GGC-3’, reverse: 5’-GCT​CTA​GAT​TTC​TGC​TCT​TTG​GT-3’, digested with *EcoRI/XbaI*, ligated with *Xba1/AscI* digested PCR product amplified from pCS105-Smad2 with the primers: forward, 5’-GCT​CTA​GAT​CGT​CCA​TCT​TGC​CAT​TCA​CG-3’, reverse, 5’-AGG​CGC​GCC​GCG​AAT​TAA​AAA​ACC​T-3’ into *EcoRI/AscI* cut pCS105 vector. For constructing *rnf2-smad2*, *rnf2* sequence was PCR-amplified with primers: forward, 5’-GGAATTC ACC​ATG​ACG​CAG​GCA​GTG​CAG​A-3’, reverse: 5’- CCG​CTC​GAG​TTT​GTG​CTC​CTT​GGT​G-3’, digested with *EcoRI/XhoI*, ligated with *XhoI/AscI* cut PCR product amplified from pCS105-Smad2 with the primers: forward, 5’-CCG​CTC​GAG​TCG​TCC​ATC​TTG​CCA​TTC​ACG-3’, reverse, 5’- AGG​CGC​GCC​GCG​AAT​TAA​AAA​ACC​T-3’ into the *EcoRI/AscI* cut pCS105 vector. RNAs were synthesized from linearized plasmids using the mMessage mMachine transcription kit (Ambion). The sequences for *rsf1* splice-blocking MO are described previously (Zhang et al., 2017). The RNAs or MOs were injected into different regions of early *Xenopus* embryos, as indicated in the text and figure legends. The doses of RNAs or MOs used are indicated in the figures and/or figure legends.

### 2.2 Whole mount *in situ* hybridization (WMISH)

Only one *rsf1* homeolog (*rsf1.S*) has been identified in *X. laevis* (XB-GENE-988573, NCBI accession # XM_018250374.2) and will be from here on in referred to as *rsf1*. The antisense digoxygenin-labeled probe for *rsf1* was made from the pBSKSII vector containing partial *Xenopus rsf1* coding sequence made by PCR cloning using the primers: forward 5’-TAC​CAG​AGC​TGC​AGG​AAG​CTG​AAG​C-3’ and reverse 5’-GAT​GTT​CTC​GAG​GCT​GAT​TCC​AAC​G-3’. The plasmid was linearized with *XhoI* (antisense) or *Pst1* (sense) and transcribed with T3 (antisense) or T7 (sense) polymerase. Sense probe labeled embryos did not display any staining (Supplementary Figure S1A). For marker gene expression analysis, MOs were co-injected with the 200 pg of RNA encoding the lineage tracer nuclear beta-galactosidase into animal regions of 2-cell stage or one dorsal animal blastomere of 4- to 8-cell stage embryos. The embryos were collected at the indicated stages, stained with the red-Gal substrate to mark the injected side, and subjected to WMISH for expression of neural, neural crest, and anterior-posterior neural patterning genes. Protocols for *in situ* hybridization and beta-galactosidase have been described (Zhang et al., 2017).

### 2.3 Immunoblot

After injection with RSF1 MO or mRNA, embryos were collected at gastrula stages and lysed in lysis buffer [50 mM Tris-HCL (pH 7.5), 150 mM NaCl, 1 mM EDTA, 10% glycerol, 0.5% Triton X-100] with 10 μl buffer per embryo at 4°C on ice. The lysate was then centrifuged at 16,000 g for 15 min and the supernatant was used for SDS-PAGE, transferred to PVDF membrane. Immunoblots were performed with anti-RSF1 (Abcam ab109002, 1:1000), anti-GFP [Santa Cruz (B-2) sc-9996, 1:600], and anti-β-actin [Cell Signaling Technology (8H110D10) # 3700, 1:2000] antibodies. LI-COR Odyssey M imager was used to detect the signals.

### 2.4 Chromatin immunoprecipitation (ChIP) assay

The ChIP assay was performed using the method as described in recent publications (Tien et al., 2021; Mohammad Parast and Chang, 2022). Briefly, the embryos were injected with 1 ng GFP-RSF1 RNA and 0.4 ng Ring1A-Smad2 or Ring1A-RQ-Smad2, cultured to the stage 11 (mid-gastrula), and harvested by cross-linking with 1% formaldehyde in 1 X PBS for about 60 min at room temperature. Sheared chromatin was collected by sonication of the embryos on ice in cold RIPA buffer, split equally into separate tubes with about 40 embryo-worth of extract in each tube, and immunoprecipitated with 1–2 μg of anti-HA (Cell signaling Technology, 3724, negative control of the experiment), anti-GFP [Santa Cruz (B-2) sc-9996], anti-H2AK119ub (Cell signaling Technology, 8240) antibodies. After reverse crosslinking and DNA extraction, qPCR was performed on an Applied Biosystems StepOnePlus cycler using PowerUp SYBR Green Master Mix (ThermoFisher A25742). The potential smad2 binding sites upstream of the *gsc* gene (*gsc.L upstream region*, accession number # AB698641.1) were analyzed by motif-based sequence analysis tool FIMO (https://meme-suite.org/meme/tools/fimo) (Grant et al., 2011). The consensus SMAD2 binding motif was extracted from JASPAR database (http://jaspar.genereg.net/) (Fornes et al., 2019). Three putative smad2 binding sites were identified around the promoter of the *gsc* gene (Supplementary Figure S3A). Two sets of primer pairs were designed to investigate the DNA immunoprecipitated by the antibodies described above (Supplementary Figure S3A). The primer sequences for ChIP-qPCR are: *gsc* primers I: forward: 5’-CTT​ACA​TTC​CCA​AAA​GAT​GAA​CAG​T-3’ and reverse: 5’-GGT​TTA​ATA​TTT​GCC​ATG​AAG​CTA​A-3’; *gsc* primers II: forward: 5’-AGA​GAA​ACA​AAA​CAG​TCA​TTC​CAT​T-3’ and reverse: 5’-ATC​TGT​GCC​TCT​TCC​CTT​ATA​TAG​C-3’. The experiment was repeated biologically three times, with each subjected to technical duplicates for qPCR. The percentage input method was used to calculate ChIP signals.

## 3 Results

### 3.1 Expression of *rsf1* during early *Xenopus* development

**FIGURE 1:**
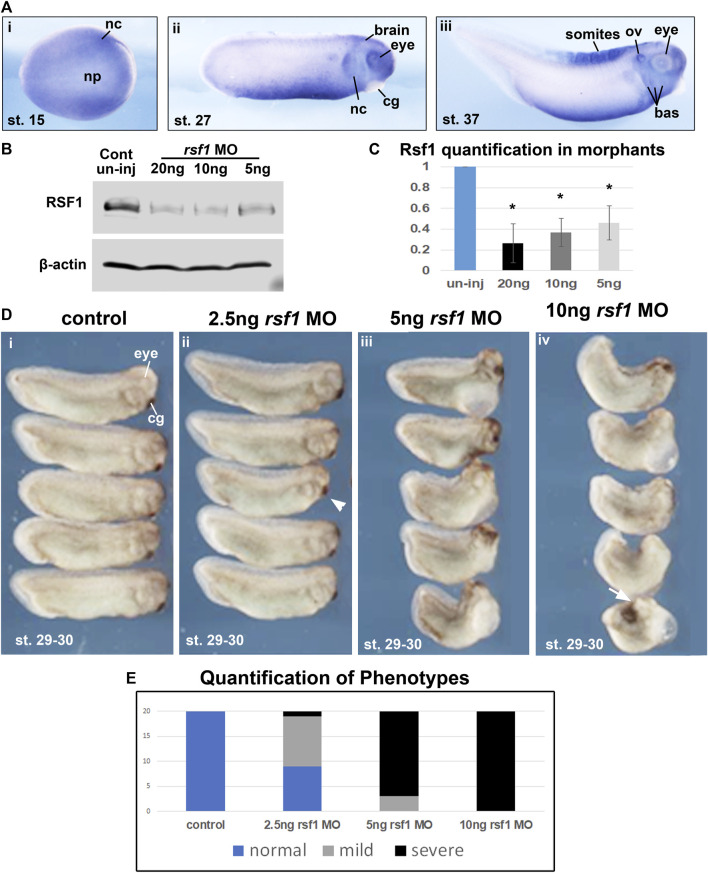
*Rsf1* is expressed in multiple tissues of *Xenopus laevis* during early development and decreasing the levels of Rsf1 causes developmental malformations. **(A)** Whole mount *in situ* hybridization of *rsf1* mRNA (purple) in *Xenopus* embryos. Representative lateral views of embryos (anterior to the right) at stages 15, 27 and 37 were shown. *Rsf1* sense probe and *rsf1* anti-sense probe with no antibody controls were included in Supplementary Figure S1A. **(B)** Immunoblots showing Rsf1 levels in control and *rsf1* MO injected embryos. The amounts of *rsf1* MO were indicated on the top of the panel, beta-actin was used as a loading control. Additional and raw images of blots are shown in Supplementary Figure S1C. **(C)** Quantification of western blots based on three replicates. One-way ANOVA revealed statistical differences among the groups (*p* < 0.001) and Holm-Sidak posthoc pair-wise comparisons showed statistical differences with each dose and control [*p* = 0.001 (uninj vs. 5 ng), *p* = 0.003 (uninj vs. 10 ng), *p* = 0.006 (ininj vs. 20 ng) SigmaPlot]. **(D)** Representative images of embryos injected with *rsf1* MO revealing malformations that were more severe with increasing concentrations. The amounts of *rsf1* MO were indicated on the top of the panel. Anterior is to the right. **(E)** Quantification of *rsf1* morphant embryos displaying various phenotypes. A mild phenotype includes small eyes, reduced head size (e.g., arrowhead). A severe phenotype includes small or no eyes, reduced head size or no head, shortened and bent body axis, failure of blastopore closure (white arrow) (*n* = 20, 2 biological replicates). nc, neural crest, np, neural plate, cg, cement gland, ov, otic vesicle, bas, branchial arches.

Our previous work demonstrated that *Xenopus rsf1*, as well as the PRC1 complex members *ring1* (*ring1*a) and *rnf2* (*ring1b*) are expressed during early embryogenesis (Zhang et al., 2017). While we have shown previously that *rsf1* has a role in mesodermal development, we also wondered if it could additionally have roles in the development of other structures. Therefore, the spatiotemporal expression of *rsf1* was examined using a whole mount *in situ* hybridization (WMISH) assay. The transcripts of *rsf1* were distributed ubiquitously during blastula and gastrula stages (Supplementary Figure S1A). At neurula stages, *rsf1* was also widely expressed through the entire embryo but additionally appeared to be enriched in a region consistent with the presumptive neural crest (Figure 1Ai). Later, the signal was further enriched in migrating neural crest, eyes, brain, and ventral mesoderm, at the tailbud stage (approximately 26 hpf at 23°C) (Figure 1Aii). After another day of development, at tadpole stages, *rsf1* also appeared in the developing branchial arches, which give rise to the craniofacial structures, and the anterior somites, which give rise to muscles and skeleton (Figure 1Aiii). The expression pattern suggests that *rsf1* may participate in the development of multiple tissues at different stages, including mesoderm, neural and neural crest.

### 3.2 Knockdown of *rsf1* induces axial defects in a dose-dependent manner

An antisense morpholino oligo (MO) that targets intron-1/exon 2 junction was designed to disrupt splicing (Supplementary Figure S1B). Consistently, when the *rsf1* MO was injected into embryos, there was a statistically significant dose-dependent reduction of Rsf1 protein levels (Figures 1B, C). Correspondingly, these doses of *rsf1* MOs also resulted in dose-dependent developmental defects (Figures 1D, E). Low doses of the MO, for the most part, induced only minor changes in embryonic morphology, including shorter length and smaller head size (Figure 1Dii, arrowhead). 85% of the embryos injected with 5 ng of the MO appeared to have more severe defects, including shortened and bent body axis as well as smaller heads (Figure 1Dii). The effects were even more severe at 10 ng where 100% of the embryos had severe malformations. In some cases, these embryos had blastopore closure defects, indicating impaired gastrulation (Figure 1Div, arrow). These effects were never observed in the sibling un-injected controls. However, to ensure the effects were not simply due to an injection effect, we also demonstrated that un-injected embryos closely resembled embryos injected with a standard control MO (Supplementary Figure S1D). Moreover, to demonstrate the specificity of the *rsf1* MO, we also determined that the defects in the Rsf1 morphants could be ameliorated by co-injecting RSF1 mRNA (Figure 3D).

**FIGURE 2:**
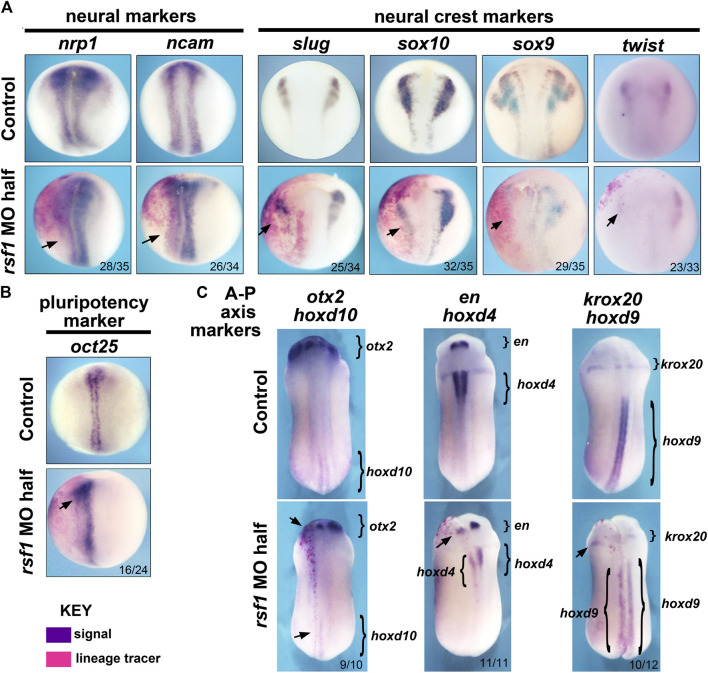
Targeted knockdown of *rsf1* in presumptive neural territories interferes with specification of neural and neural crest tissues and affects anterior-posterior neural patterning. Embryos were all injected with 2.5 ng of MO into one dorsal blastomere. **(A)** Targeted knockdown of Rsf1 in presumptive neural territories downregulated neural and neural crest marker genes. Dorsal views of representative embryos at stage 17, anterior to the top and the injected side is on the left. The neural (*nrp1* and *ncam*) and neural crest (*slug*, *sox10*, *sox9*, and *twist*) markers were reduced in the embryos on the side of targeted injection. 100% of controls had expression pattern similar to the representative embryos shown (black arrows, *n* = 33–35, 2 biological replicates for each marker). **(B)** The pluripotent marker gene *oct25* was expanded on the *rsf1* MO injected side. 100% of controls had expression pattern similar to the representative embryos shown (*n* = 24, 2 biological replicates). **(C)** Targeted knockdown of *rsf1* in presumptive neural territories affected A-P axis gene expression. Dorsal views of representative embryos at stage 24, anterior to the top and the injected side is on the left. Bracketed regions indicate expression domains, and black arrows point to areas where there is a reduced expression. The numbers of the morphant embryos with expression pattern changes similar to those shown are indicated in the bottom right of each panel. 100% of controls had expression pattern similar to the representative embryos shown (*n* = 10–12, 2 biological replicates).

Previously, we noted the Rsf1 has a role in mesodermal development (Zhang et al., 2017). Consistently, we observed expression of *rsf1* throughout the gastrula stages, including the presumptive mesoderm, as well as gastrulation-type defects in the morphants. Additionally, *rsf1* expression was also observed in neural and neural crest tissues and the *rsf1* morphants had head defects (Figure 1A). These data prompted us to further investigate the role for Rsf1 in the specification of these tissues. To do so, we performed targeted injections of the MO and determined how this would affect the expression of genes that not only mark neural and neural crest but also regulate of neural or neural crest development. In these experiments, *rsf1*-MO was co-injected with an RNA lineage tracer that encodes nuclear β-galactosidase. Both the MOs and lineage tracers were injected into the animal region of one dorsal blastomere of four-to eight-cell stage embryos. As a result, these reagents would be localized to one half of the embryo in tissues fated to become the neural and neural crest. The embryos were collected at early stages when the neural and neural crest are specified. Then they were stained with a substrate of β-galactosidase (red-Gal) to mark the injected side, and subjected to WMISH for expression of neural and neural crest markers. We found that the pan-neural genes *nrp1* and *ncam* were slightly reduced, especially in the posterior regions of 80% and 76% of the embryos respectively (Figure 2A). Decreased Rsf1 resulted in an even more dramatic reduction in neural crest marker genes *slug*, *sox10*, *sox9*, and *twist* on the MO-injected side when compared with that in the un-injected side (70%–91%, Figure 2A). Another important modulator of neural development is OCT4. This protein is thought to be integral to maintaining cell pluripotency and delay cell differentiation (Tien et al., 2021). Therefore, we examined the expression of the *Xenopus* OCT4 ortholog *pou5f3.2*, also called *oct25*. Results showed that the expression of this gene was expanded by Rsf1 knockdown in 67% of the morphant embryos (Figure 2B). These results point to a hypothesis where Rsf1-mediated PRC1 function is required for repressing pluripotency in the neural and neural crest domains to allow proper cell differentiation of these tissues.

PRC1 has also been shown to regulate anterior-posterior (A-P) body patterning in both invertebrates and vertebrates, at least partially *via* its ability to regulate its canonical targets, the *hox* genes (Soshnikova, 2014; Gentile and Kmita, 2020). To investigate whether Rsf1 shares this function with PRC1, specifically in the developing nervous system, we again injected *rsf1* MO unilaterally into the animal region of one dorsal blastomere of four- to eight-cell stage embryos with the lineage tracer as described above. Marker gene expression was analyzed at approximately 24 hpf at 23°C when the A-P axis of the brain is forming. Analysis of the anterior neural patterning genes *otx2* and *engrailed (en)* revealed that *rsf1* MO reduced the expression of these genes in the head (Figure 2C, arrows). Moreover, decreasing the levels of Rsf1 also caused a posterior shift in the expression domains of *krox20*, *hoxd10*, *hoxd4*, and *hoxd9* on the MO-injected side of the embryos (Figure 2C). Collectively, these data suggest that Rsf1 indeed has a role in the patterning of the A-P axis of the nervous system.

### 3.3 The UAB domain is critical for the function of Rsf1 in *Xenopus* development

**FIGURE 3:**
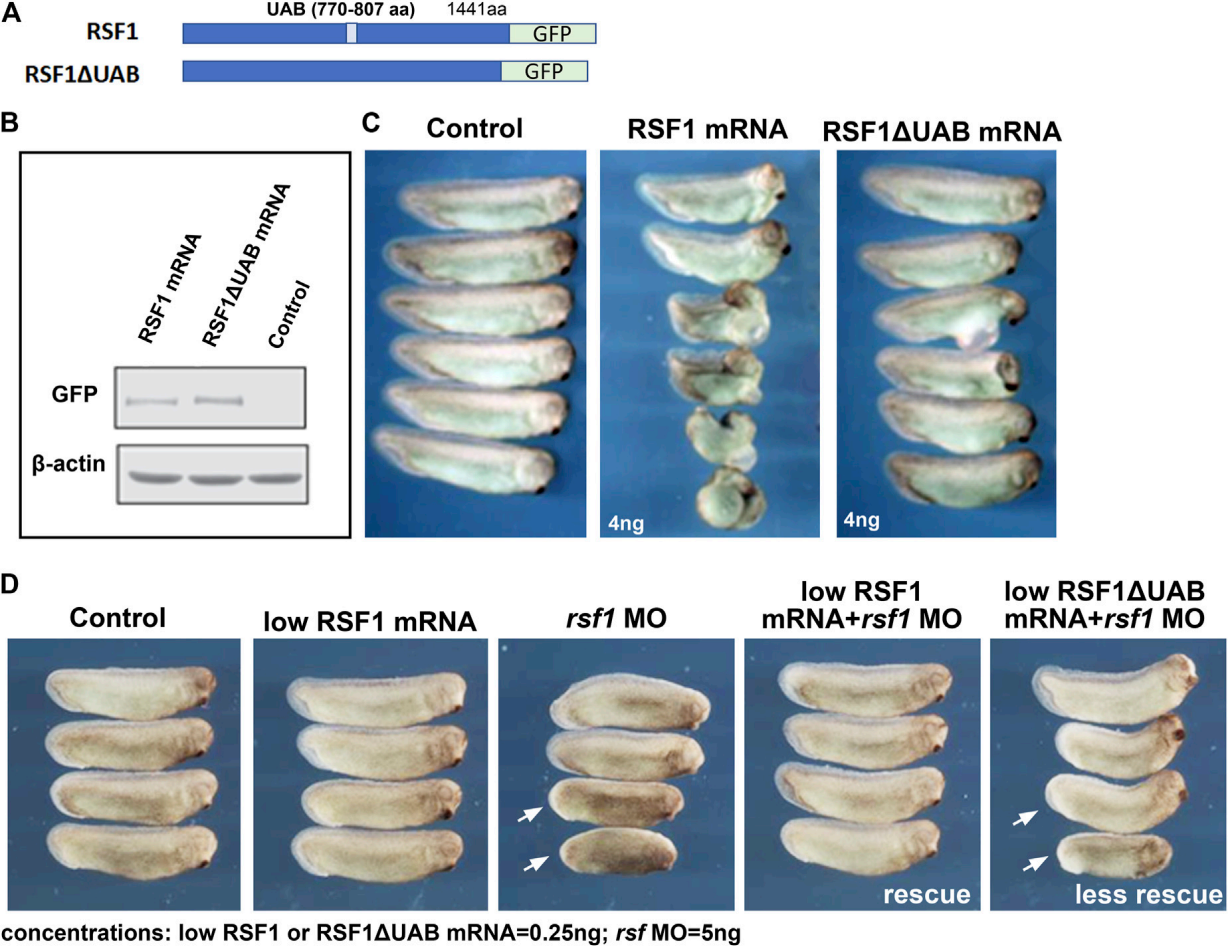
The UAB domain is required for RSF1 to regulate *Xenopus* embryonic development. **(A)** Schematic representation of the two constructs used in the study consisting of a human RSF fused to GFP and a mutant form of RSF1 with the UAB region deleted and fused to GFP. **(B)** Immunoblots of RSF protein in extracts prepared from *Xenopus* embryos injected with RSF1 or RSF1ΔUAB mRNA. The levels of GFP fusion proteins appear the same. β-actin was used as loading controls. Additional images and raw data are included in Supplementary Figure S2A. **(C)** Embryos injected with 4 ng of RSF1 mRNA resulted in major malformations. However, much less severe developmental defects were observed in embryos injected with 4 ng RSF1ΔUAB mRNA. Representative embryos are shown with anterior to the right (*n* = 18, two biological replicates). **(D)** RSF1 but not UAB deleted RSF1ΔUAB rescued the developmental defects caused by *rsf1* knockdown. The malformations induced by 5 ng of rsf*1* MO were partially rescued by co-injection of low concentrations (0.25 ng) of RSF1 mRNA but not 0.25 ng of RSFΔUAB mRNA (compare embryos with white arrows). Control embryos are un-injected siblings (*n* = 20, 2 biological replicates). Four representative embryos are shown with anterior to the right.

The UAB domain in RSF1 was identified in our previous studies as a crucial region for binding to H2AK119ub nucleosomes and mediates its repressive activity on gene expression (Zhang et al., 2017). To determine whether the UAB domain is important for RSF1 to regulate *Xenopus* development, we examined the effects of wild-type human RSF1 and the UAB domain-deleted mutant (RSF1ΔUAB) on *Xenopus* embryogenesis. These sequences were fused with GFP to serve as a tracer (Figure 3A). Capped mRNA generated from these constructs were injected into the dorsal marginal zone of 4-cell stage embryos, and the effects on embryogenesis were tested at a variety of concentrations (*not shown*). We determined that 4 ng of RSF1 mRNA produced a significant defect. The levels of ectopically expressed RSF1 or RSF1ΔUAB proteins were similar in embryos injected with this concentration of mRNA (Figure 3B). Embryos injected with RSF1 mRNA had moderate-to-severe defects, such as curved body axis, smaller heads, and gastrulation defects (Figure 3C). In contrast, injection of RSF1ΔUAB mRNA in sibling embryos resulted in much less severe defects (Figure 3C). The notable difference in phenotype severity upon expression of similar levels of RSF1 and RSF1ΔUAB suggests that the UAB domain is important for the function of RSF1 during *Xenopus* development (Figure 3C).

To further confirm the importance of the UAB domain for the function of RSF1 in development, we compared whether RSF1ΔUAB mRNA could ameliorate the effects of *rsf1* MO knockdown as effectively as wild-type RSF1 mRNA. For this purpose, we titrated down the concentrations of RSF1 mRNA or RSF1ΔUAB mRNA, so that no obvious malformations could be observed (0.25 ng/embryo, Figure 3D). We injected a moderate dose of *rsf1* MO (5 ng/embryo) alone and observed a shortened body axis induced in approximately 50% of the embryos. Using the sub-phenotypic dose of RSF1 mRNA in combination with a moderate dose of *rsf1* MO, we observed that this shortened body axis could be rescued (Figure 3D). In contrast, a sub-phenotypic dose of RSF1ΔUAB mRNA did not rescue the *rsf1* MO effects as effectively (Figure 3D). Taken together, these data suggest that the UAB domain is important for the function of RSF1 and that recognition of H2AK119ub may underlie the activity of Rsf1 to regulate *Xenopus* embryogenesis. Furthermore, these experiments also demonstrate the specificity of the *rsf1* MO as well as the conservation of Rsf1 function, since we used human RSF1 sequences.

### 3.4 Fusion of *ring1a* to *smad2* limits the function of *smad2* in mesoderm induction

**FIGURE 4:**
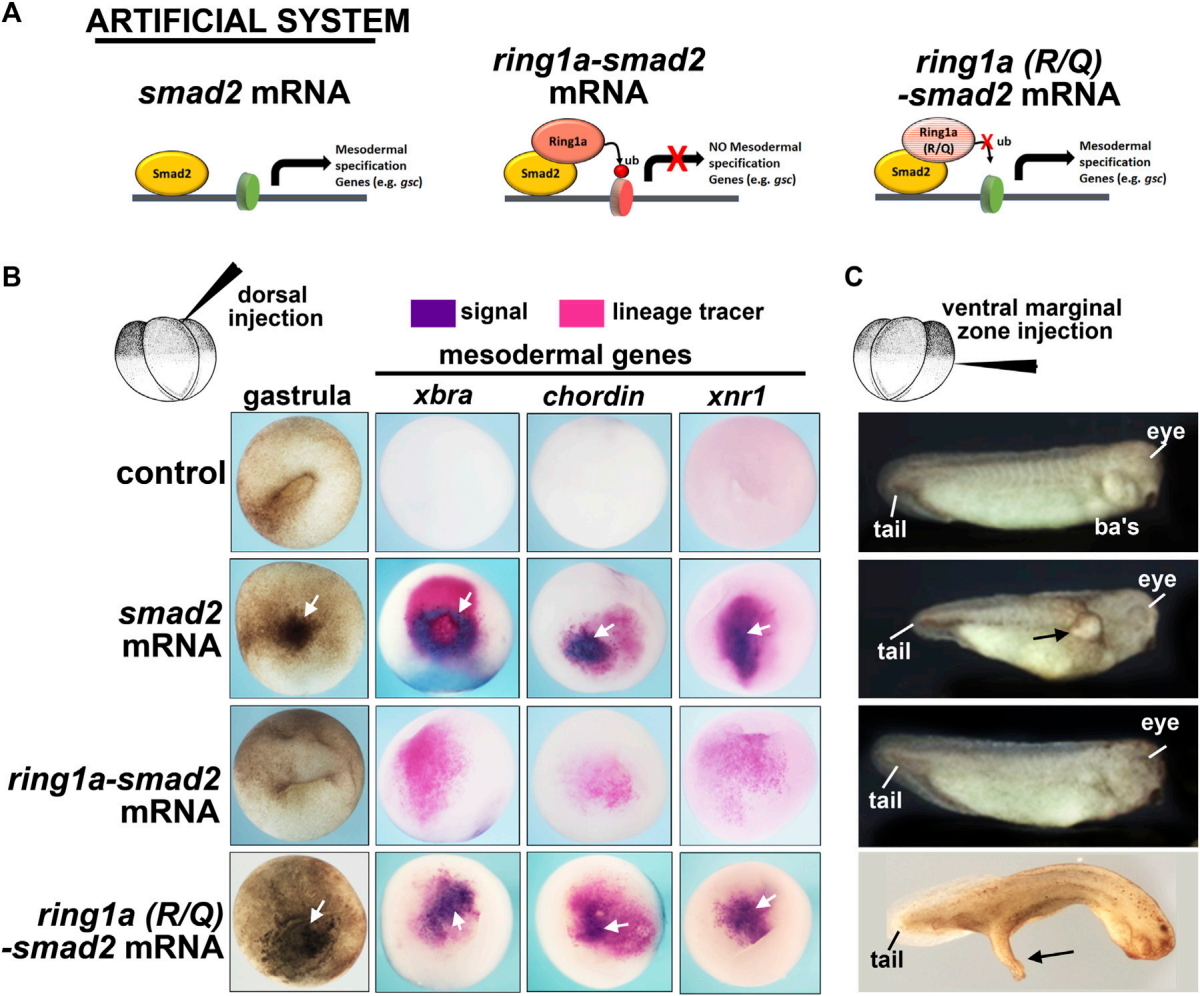
Fusion of *ring1a* to *smad2* limits the function of *smad2* in mesoderm induction. **(A)** Schematic showing the experimental system. **(B)** Embryos dorsally injected with (0.25 ng) of mRNA were examined for changes in pigmentation during gastrulation and inappropriate expression of mesodermal genes, all of which indicate mesoderm induction. Additional pigment and mesodermal gene expression are indicated by white arrows (*n* = 19–20, two biological replicates). **(C)** Embryos ventrally injected with (0.25 ng) of mRNA were examined for protrusions, indicatives of secondary axis formation. Overt protrusions are indicated by black arrows. *N* = 18–20, two biological replicates. ba’s, branchial arches.

Our next goal was to test the hypothesis that Rsf1 mediates the gene repressive effects of H2AK119ub during PRC1-regulated embryonic development. To do this, we created an artificial system that would generate ectopic H2AK119ub marks on target genes. We then used this system to examine the role of Rsf1 in regulating the expression at the ectopic gene locus (see the next section). Our artificial system consisted of the PRC1 subunit Ring1a, which we have shown in our previous work to play major roles in *Xenopus* development (Zhang et al., 2017). The Ring1a protein was fused to Smad2, which is a well-studied signaling molecule acting downstream of the mesodermal inducing Nodal (Schier, 2003). The prediction was that Ring1a, when fused to Smad2, would deposit ubiquitin marks at histone H2AK119 on target genes and these genes would then be repressed rather than induced (Figure 4A).

We first tested this fusion construct on mesoderm development *in vivo* using our *Xenopus* model. When *smad2* is expressed in the animal region, it induced ectopic mesoderm, as visualized by dark pigmented cells indicative of ectopic bottle cells at gastrula stages (Schier, 2003). Indeed, *smad2* mRNA injected embryos appeared to have abundant pigmented cells in the ectodermal region (Figure 4B, white arrow). On the other hand, *ring1a-smad2* mRNA was less effective in inducing such ectopic pigmented cells, indicating a reduction in mesoderm induction (Figure 4B). To further assess mesoderm development, we next examined the expression of mesodermal marker genes, which are expressed in the early mesoderm. This analysis revealed that while *smad2* mRNA induced the expression of the mesodermal markers *brachyury* (*xbra*), *chordin*, and *xnr1*, the *ring1a-smad2* mRNA failed to induce these markers (Figure 4B). To ensure that the effect of Ring1a-Smad2 fusion protein on mesoderm induction was specific, we performed additional controls. First, *ring1a* mRNA alone was injected into embryos and it was determined that Ring1a alone did not induce excess pigmentation at gastrula stages, nor did it induce secondary axis formation (Supplementary Figure S2B). One might argue that the artificial fusion construct might affect mesodermal gene expression due to the steric hindrance of the added Ring1a. Therefore, we included another control, in which an R to Q point mutation in the catalytic domain of the Ring1a protein that abolishes its ubiquitin ligase activity was introduced into the *ring1a-smad2* construct. Embryos injected with *ring1a(RQ)-smad2* RNA appeared similar to embryos injected with *smad2* mRNA alone in inducing pigmented cells and expression of mesodermal markers (Figure 4B). These results indicate that the fusion protein likely acted as predicted and its repressive effects on mesoderm induction required the H2AK119ub activity of Ring1a. Finally, we also constructed a fusion of Smad2 with the other PRC1 complex member Ring1b (also called Rnf2 in *Xenopus*). Results revealed that embryos injected with *smad2-rnf2* mRNA did not abolish the Smad2-induced expression of *xbra*, however, it did reduce the expression of mesodermal markers *chordin* and *xnr1* (Supplementary Figure S2C). These results suggest that Ring1a is the primary regulator of *Xenopus* mesodermal development, consistent with previous reports (Zhang et al., 2017).

Overexpression of *smad2* can also induce ectopic secondary axis formation when expressed in the ventral region (Schier, 2003). Indeed, when we injected *smad2* RNA in the ventral marginal zone of 4-cell stage embryos, we observed the formation of partial secondary axis as a large protrusion (Figure 4C). However, expression of the *ring1a-smad2* mRNA in the ventral region did not induce these protrusions as effectively, some were absent or much smaller (Figure 4C). Embryos injected with *ring1a(R/Q)-smad2* RNA, however, developed secondary axis protrusions, similar to the *smad2* mRNA alone (Figure 4C). These results thus validate our artificial system, showing that the Smad2-Ring1a fusion has repressive properties that rely on the Ring1a ubiquitin ligase activity. These results are consistent with our hypothesis that Ring1a is recruited to the Smad2 target genes by the fusion protein, deposits ectopic H2AK119ub marks at the regulatory regions of these genes, and leads to inefficient activation or even repression of Smad2 transcriptional activity (Figure 4A).

### 3.5 Ring1a-Smad2 deposits H2AK119ub marks at a Smad2 target gene and recruits RSF1

**FIGURE 5:**
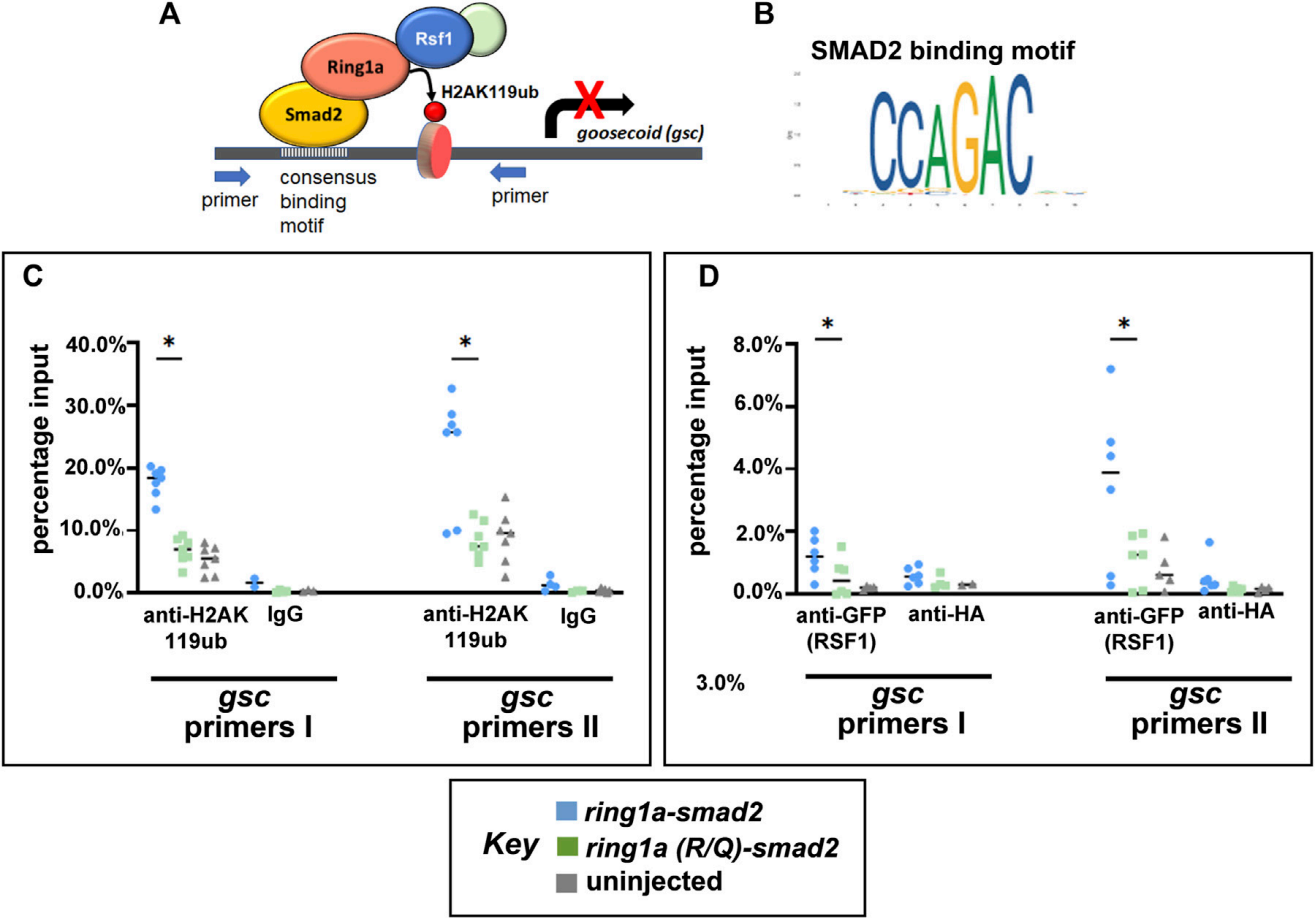
*Ring1a-smad2* deposits H2AK119ub marks on the *smad2* target gene *gsc* and recruits RSF1. **(A)** Schematic of the experimental hypothesis. **(B)** SMAD2 binding motif. **(C)** Real-time PCR of input and chromatin immunoprecipitated DNA by the indicated antibody. Embryos were co-injected with GFP-RSF1 (1 ng) and *ring1a-smad2* or *ring1a-R/Q-smad2* (0.4 ng) and subjected to immunoprecipitation using anti-H2AK119ub antibody. IgG was used as negative controls. ChIP signals were normalized to input signals and percentage inputs were plotted and shown for two primer pairs. **(D)**. Real-time PCR of input and chromatin immunoprecipitated DNA by the indicated antibodies. Embryos were injected as in **(C)** and subjected to immunoprecipitation using anti-GFP antibody. Anti-HA antibody was used as negative controls. Percentage input results from each technical repeat are represented by symbols and median is shown as a short bar. Wilcoxon matched-pairs signed rank test revealed statistical differences between *ring1a-smad2* and *ring1a(R/Q)-smad2* groups (*p* < 0.05). Raw data were included in Supplementary Figure S3B.

Now that we have established an artificial system to test for Ring1a-dependent H2AK119ub activity and Rsf1 recruitment *in vivo*, we next asked whether Ring1a-Smad2 indeed deposits ectopic H2AK119ub marks directly to a Smad2 target and then recruits RSF1 for gene repression (Figure 5A). To do this, we chose a potential direct target of Smad2 in *Xenopus* development, the *gooscoid* (*gsc*) gene (Kim et al., 2011). This gene has also been well established to be an integral regulator of mesoderm induction and organizer formation (Sudou et al., 2012). To identify the Smad2 binding sites in *Xenopus gsc*, we first used JASPAR, an online open-access database storing manually curated transcription factor binding profiles (Fornes et al., 2019). This database identified a SMAD2 consensus binding sequence CCAGAC (Figure 5B). We then searched the promoter regions of the *Xenopus gsc* gene using the online motif-searching tool FIMO (Grant et al., 2011). The result revealed three Smad2 consensus motifs within the promoter of this gene and primers were designed to flank these motifs (Supplementary Figure S3A). Chromatin immunoprecipitation (ChIP) assay with anti-H2AK119ub antibody revealed that embryos injected with *ring1a-smad2* mRNA had enhanced H2AK119ub signals at the *gsc* gene promoter (Figure 5C), as compared with embryos injected with mutant *ring1a(R/Q)-smad2* mRNA (Figure 5C).

To explore whether ectopic H2AK119ub marks could cause the recruitment of the RSF1 protein, we also performed ChIP against GFP in embryos co-injected with GFP-RSF1 and *ring1a-smad2* or *ring1a(R/Q)-smad2* mRNA. We observed that embryos injected with *ring1a-smad2* indeed showed higher signal for GFP (indicative of RSF1 binding) at the *gcs* promoter regions when compared with embryos injected with *ring1a(RQ)-smad2* (Figure 5D). In these experiments, we included an uninjected group as negative control and normalized our ChIP data against uninjected group signals (Figures 5C, D). The data provide direct evidence that the PRC1-H2Aub-RSF1 system, which was originally identified in biochemical and cell-based assay, is also operating *in vivo* during vertebrate development.

## 4 Discussion

RSF1 was first identified as the non-catalytic subunit of RSF complex, together with the ATP-dependent subunit SNF2H, to facilitate activator-dependent transcription initiation on chromatin templates (LeRoy et al., 1998). Subsequently, RSF was shown to mediate nucleosome deposition and generate regularly spaced nucleosome arrays in an ATP-dependent manner (Loyola et al., 2001; Loyola et al., 2003). In *Drosophila*, mutant Rsf-1 behaved as a dominant suppressor of position effect variegation (Hanai et al., 2008). Kdm2, a subunit of the variant PRC1.1 complex, specifically pulled down CG8677, the *Dosohplia* homolog of RSF1 (Kang et al., 2022). Together, these data suggested that RSF1 may be a missing factor in the PcG system. Indeed, our previous work showed that RSF1 can recognize H2AK119ub nucleosomes and is involved in PRC1-mediated gene repression (Zhang et al., 2017).

PcG proteins are essential for maintaining cell type-specific gene expression programs and, therefore, cell identity (Boyer et al., 2006; Bracken et al., 2006; Lee et al., 2006; Thornton et al., 2014). For example, mouse embryonic stem cells lacking PcG proteins such as EZH2, SUZ12, EED and Ring1B, are often unstable and tend to differentiate spontaneously (Pasini et al., 2007; Chamberlain et al., 2008; Voncken et al., 2003; O'Carroll et al., 2001). When induced to differentiate, these knockout ESC lines also fail to undergo successful lineage commitment, demonstrating a clear role for PcG proteins in cell fate. Since PcG complexes such as PCR1 are critical for defining cell identity and RSF1 is required for PRC1-mediated gene repression, it was an obvious next step to explore a role for RSF1 in embryonic development. Understanding how RSF1 functions in the embryo has the potential to begin to fill a gap in our understanding of gene repression during specification of cell fates. However, PcG protein knockout mice die at the embryonic stage after implantation but before finishing gastrulation. The early lethality prevents further dissection of the function of PcG protein in mammalian embryogenesis. Therefore, we have turned to another vertebrate model, *X. laevis.* This model has been widely used for studying epigenetic modulators during development (Bajpai et al., 2010; Tahir et al., 2014; Greenberg et al., 2019; Wyatt et al., 2021; Mohammad Parast and Chang, 2022) and has several advantages (Bowes et al., 2008; Tandon et al., 2017). For example, *Xenopus* embryos are free-living, develop rapidly, and can be obtained simultaneously (by *in vitro* fertilization) in great numbers with synchronous development. This allows us to monitor changes in development in real time and is ideal for biochemical analyses. In addition, cell fates have been mapped to early cleavage stage embryos (Moody, 1987; Moody, 2019), thus allowing targeted injection of reagents into defined regions to specifically affect gene levels in tissue-specific manners. Importantly, tools to modify levels of gene expression are titratable and thus we can avoid the lethality of gene knockout, which occur in mouse models (Eisen and Smith, 2008).

Here, using *X. laevis* as a model, we examined the PRC1-H2AK119ub-RSF1 pathway in embryogenesis. One major goal was to demonstrate that this system worked in a similar way during development. We have previously defined a specific domain, called UAB, in RSF1 that can recognize H2AK119ub nucleosomes. Here, we demonstrated that this domain is indeed critical for RSF1 function in embryos as well. This provides additional evidence that this is a conserved pathway. Importantly, we also determined that, like in mammalian cells, RSF1 is recruited to PRC1-mediated H2AK119ub. To show this, we created an artificial system using the known role of Smad2 in mesoderm development. Smad2 is activated by Nodal signaling during induction of mesoderm during gastrulation (Schier, 2003). For this reason, ectopic expression of Smad2 in the animal region (where ectoderm forms) results in the inappropriate induction of mesoderm. As we expected, when Smad2 was fused to Ring1a, mesoderm was no longer induced, suggesting that Ring1a deposited the repressive H2AK119ub marks on genes required for mesodermal development. This was not due to *ring1a* expression nor due to the steric hindrance of the fused proteins, since a point mutation to *ring1a* did not have the same repressive effect. Similarly, when *smad2* is overexpressed in a ventral blastomere, embryos form a secondary axis due to additional and inappropriate organizer induction (Schier, 2003). Again, as expected, the Smad2-Ring1a fusion could repress this developmental anomaly. Next, we used this system to directly examine changes in H2AK119ub marks on consensus Smad2 binding motifs in a Smad2 target gene, *gsc*. The ChIP analysis demonstrated that the Smad2-Ring1 fusion protein deposited H2AK119ub marks. Importantly, the epitope H2AK119ub marks recruit RSF1. Thus, these results support our model established with cell-based assays (Figure 5A).

In previous work, we revealed a role for Rsf1 in gastrulation and mesoderm development (Zhang et al., 2017). In the present study, we extended the data to reveal additional roles for Rsf1 in neural and neural crest development. Specification of each of these tissues requires their own sophisticated orchestration of signaling and transcriptional regulators [reviewed in (Sargent, 2006; Prasad et al., 2012; Mayor and Theveneau, 2013; Bronner and Simões-Costa, 2016)]. Targeted injection of *rsf1* antisense oligonucleotides (morpholinos) into blastomeres that are fated to the neural and neural crest progenitors resulted in a reduction in the expression of markers of these tissues, which are also critical for their development. These results are consistent with *rsf1* morphant embryos having a smaller head, possibly due to the failure of brain and cranial neural crest cell proliferation and differentiation. Interestingly, we also observed an expansion in *oct25*, the *Xenopus* ortholog of OCT4. It has been proposed that OCT4 has been co-opted by neurons and neural crest cells to expand their developmental potential and maintain a multipotent state (Patel and Parchem, 2022). Future work in dissecting the role of PRC1-H2AK119ub-RSF1 regulation of gene expression could provide a better understanding of the balance between pluripotency and differentiation during both neural and crest development. PcG proteins are also known to regulate homeobox transcription factors (Gentile and Kmita, 2020). These proteins are critical regulators of cell identity and positioning in embryonic development, specifically in the nervous system (Soshnikova, 2014; Parker et al., 2018; Saito and Suzuki, 2020). In this study, Rsf1 knockdown resulted in posterior shifts in the expression of two *Hox* genes and the homeobox-containing gene *krox20*, all expressed in the developing nervous system. Our data suggests that Rsf1 could also be important for delineating embryonic *hox* gene expression domains.

In conclusion, based on the work presented here and in previous studies (Zhang et al., 2017), we propose that RSF1 is an integral component of the PRC1-H2AK119ub epigenetic system. Importantly, we solidify that the PRC1-H2AK119ub-RSF1 system is also operating in the specification of several fundamental tissue types in vertebrate embryonic development.

## Data Availability

The raw data supporting the conclusion of this article will be made available by the authors, without undue reservation.
