# Supplementary material for: Recognition of H2AK119ub plays an important role in RSF1-regulated early Xenopus development
Source: Front Cell Dev Biol. 2023 Jul 17;11:1168643. doi: 10.3389/fcell.2023.1168643 (PMC10389277; doi:10.3389/fcell.2023.1168643)
Supplement: Supplementary file 1 [file DataSheet1.PDF]

Supplementary Material

Recognition of H2AK119ub plays an important role in RSF1-regulated early Xenopus development

Saeid Mohammad Parast, Deli Yu, Chunxu Chen, Amanda Dickinson, Chenbei Chang, Hengbin Wang*

*** Correspondence:** Hengbin Wang: hengbin.wang@vcuhealth.org

1 Supplementary Figures and Tables

This Supplemental file contains three Supplemental Figures with Figure Legends.

Supplemental Figure 1

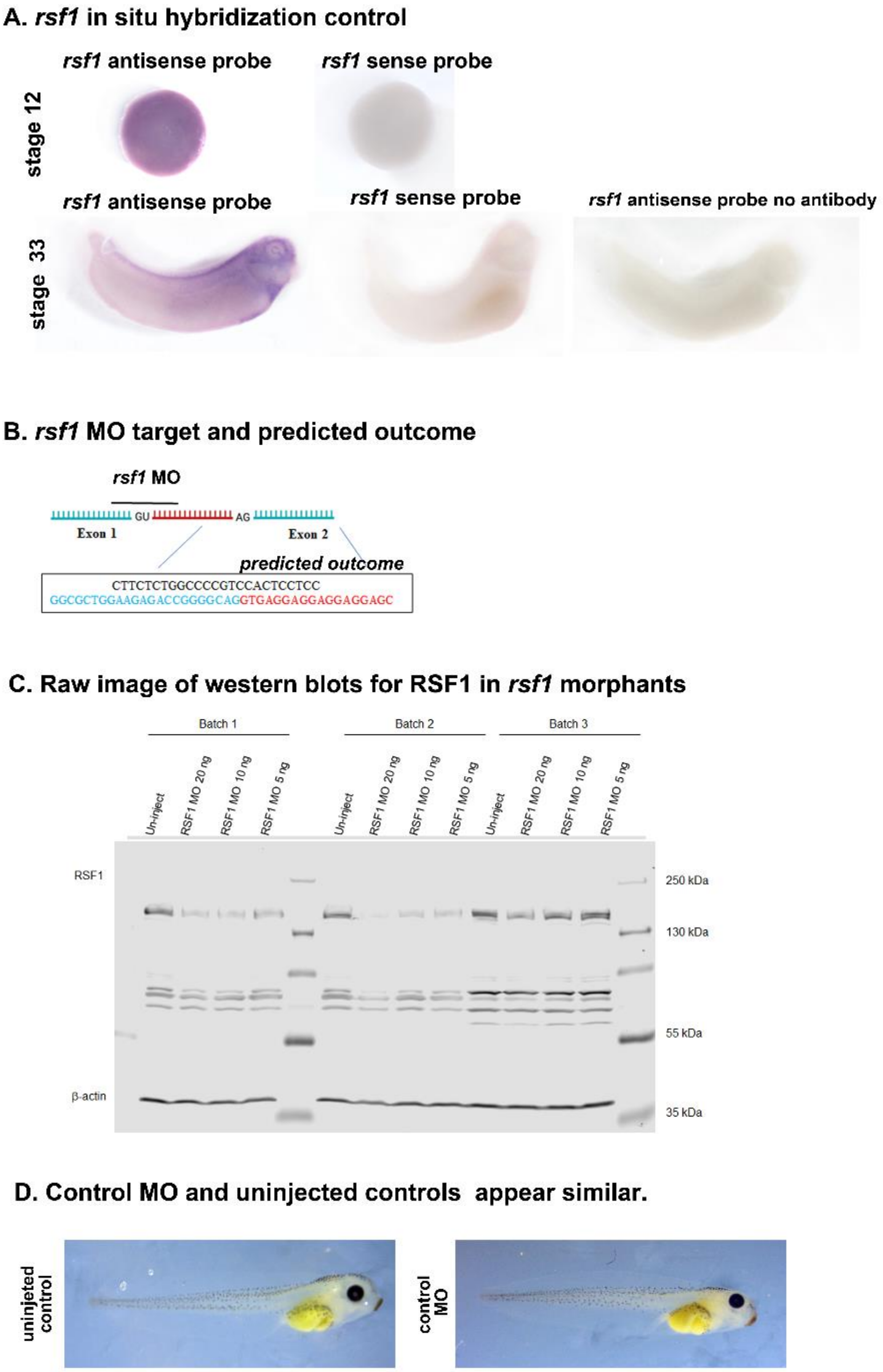

Figure S1: A) In situ hybridization showing sense and no antibody controls. **B)** Schematic showing that the antisense *rsf1* MO was designed to target the splice donor site between intron 1 and exon 2, which is expected to cause retention of intron 1 and introducing a stop codon to produce a truncated protein with only 98 amino acids. **C)** Raw images of Western Blots of Rsf1 in *rsf1* morphant embryos with beta-actin as a loading control. **D)** Lateral views of representative sibling embryos either injected with a standard control MO or uninjected. This image shows that 60 ng of control MO (well exceeding the amounts used in this study) do not cause developmental abnormalities.

Supplemental Figure 2

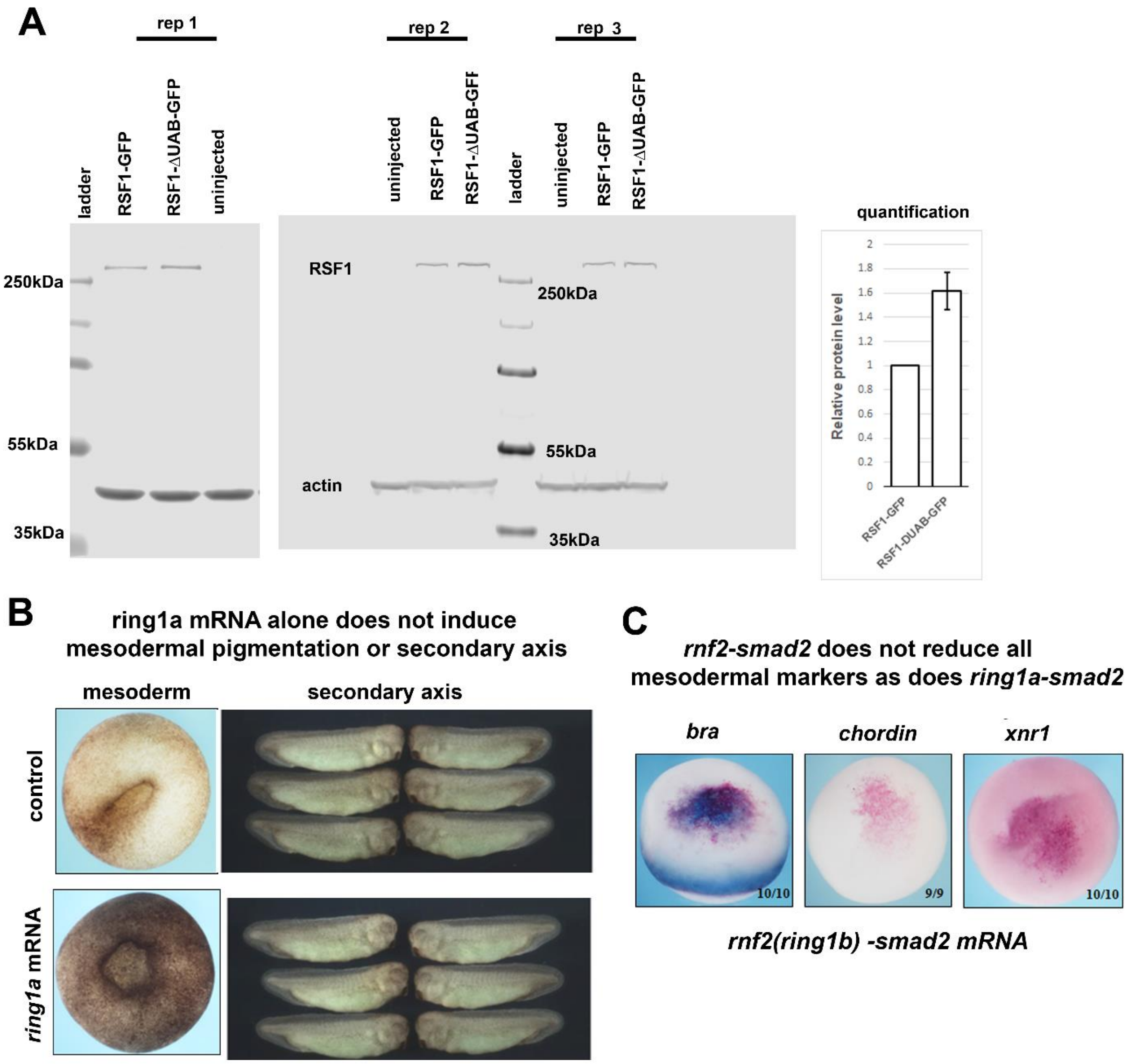

Figure S2: A) Raw images and additional experiments of Western Blots of GFP-RSF1 and GFP-RSF1ΔUAB in injected embryos with beta-actin as a loading control. Quantification of western blots based on the three replicates is shown in the right. **B)** Injection of *ring1a* mRNA alone does not induce pigmentation (indicative of mesodermal induction), nor secondary axis. **C)** *rnf2-smad2* mRNA injections reduce the mesodermal marker *chordin* and *xnr1* but not *bra,* suggesting that its effects on mesodermal gene expression are less effective as *ring1a-smad2*.

Supplemental Figure 3

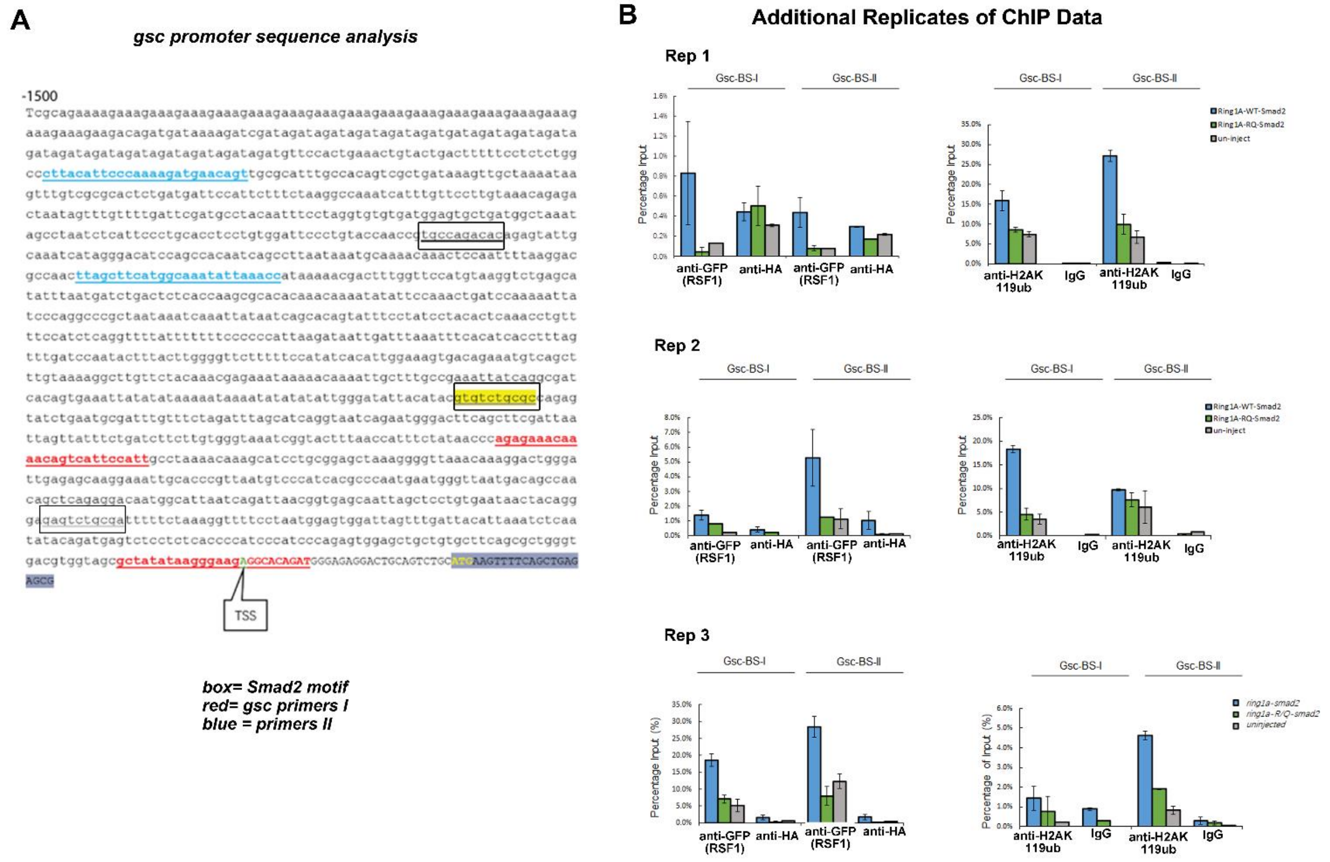

Figure S3. A) Sequence analysis of the upstream regulatory region of the *gsc* gene. Computational analyses of the *gsc* regulatory region reveal several putative Smad2 binding motifs, the top three are shown here (in boxes and underlined, the darker the higher the score). Primers used to amplify the indicated regions are shown in blue (*gsc* primers I) and red (*gsc* primers II). Transcription start site (TSS) is indicated in green and by a box. Protein coding sequence is highlighted in grey. **B)** Raw data of the three times ChIP-qPCR.
